# Lactylation-related genes signature panel in hepatocellular carcinoma reveals the prognostic and therapeutic optimization

**DOI:** 10.1080/23723556.2026.2621475

**Published:** 2026-02-22

**Authors:** Ting Xiao, Xianqun Xu, Ziwu Zhao, Han Xue, Shuang Guo, Xinghua Long

**Affiliations:** a Department of Laboratory Medicine, Zhongnan Hospital of Wuhan University, Wuhan, China

**Keywords:** Lactylation, bioinformatics, cancer, diagnosis, therapy

## Abstract

Lactylation-related genes (LRGs) exert a significant influence on tumor progression and are critically involved in modulating responses to immune checkpoint inhibitors (ICIs). Leveraging a curated set of 332 LRGs derived from the literature, we identified prognosis-specific hepatocellular carcinoma (HCC) clusters through consensus clustering analysis. Using an integrated bioinformatics approach, we systematically evaluated differences in immune microenvironment infiltration, somatic mutations, copy number variations, and epigenetic modifications between the two tumor subtypes. We subsequently developed a lactylation-related genes signature (LRGS) and independently validated its prognostic efficacy in the TCGA cohort. Notably, the high-LRGS group exhibited significantly reduced overall survival(OS) and diminished response to ICIs. Conversely, the high-LRGS group demonstrated heightened sensitivity to several antitumor agents, including AZD5153, cediranib, foretinib, and vorinostat, relative to the low-LRGS group. Our findings establish a robust LRGS model, offering a clinically actionable tool for prognostic assessment and precision therapy in HCC.

## 1 Introduction

Hepatocellular carcinoma (HCC) is a leading cause of global cancer mortality, characterized by aggressive invasion and therapeutic resistance that present major clinical challenges.^1^^,^^2^ Although targeted therapies (e.g., sorafenib, lenvatinib) and immune checkpoint inhibitors (ICIs) have significantly improved clinical outcomes, the etiology of tumors is highly complex and heterogeneous.^3^ It encompasses not only driver gene mutations and chromosomal instability at the genetic level, but also epigenetic regulatory disorders, tumor microenvironment remodeling, and the effects of predisposing factors such as viral infection.^4^^,^^5^ This multifactorial and intertwined etiological feature directly leads to significant differences in treatment response, disease progression rate, and survival prognosis among different patients. This underscores the urgent need for predictive biomarkers that enable personalized, cost-effective ICI-based treatment strategies.

Recent advances have highlighted the importance of protein posttranslational modifications—including lactylation, acetylation, and ubiquitination—in driving tumor progression through the dynamic regulation of signaling pathways, metabolic reprogramming, and epigenetic remodeling.^6^^,^^7^ Among these, lactylation, a metabolism-linked modification, has emerged as a key regulator of cellular energy metabolism and the immune microenvironment. A hallmark of tumor cell metabolism is the Warburg effect, wherein cancer cells preferentially metabolize glucose via aerobic glycolysis, even under oxygen-rich conditions, resulting in excessive lactate accumulation.^8^^,^^9^ Contrary to the traditional view of lactate as a metabolic byproduct, emerging evidence indicates that it functions as a signaling molecule, modulating the cell cycle and tumor proliferation.^10^ Moreover, lactate reprograms CD4^+^ T cell metabolism and epigenetics to promote pro-inflammatory polarization.^11^ In the tumor microenvironment, lactate preferentially fuels regulatory T cell energy metabolism and immunosuppressive function through MCT1/LDHB-dependent pathways, while simultaneously impairing effector T cell activity to facilitate immune evasion.^12^

Hallmark features of HCC, including enhanced glycolysis and lactic acid accumulation, may provide the microenvironmental basis for lactylation modifications.^13^ Yang et al. found that lysine lactylation (Kla) from cytosolic lactate was positively correlated with the concentration of cytosolic lactate, and that lactylation of the K28 site inhibited the function of adenylate kinase 2 and promoted the proliferation and metastasis of HCC cells.^14^ Jin et al. found that lactylation of CCNE2 promoted HCC cell growth, whereas SIRT3 catalyzed de-lactylation of CCNE2, thereby inhibiting HCC cell proliferation, migration, and invasion.^15^ In addition, dysregulated lactate metabolism drives immune dysfunction through three interconnected mechanisms: direct metabolic competition, acidotic suppression of immune cells, and lactylation-mediated epigenetic reprogramming of key signaling pathways in HCC.^16^ Lactylation contributes significantly to HCC progression and represents a potential therapeutic obstacle. Previous studies have shown that lactylation-related genes (LRGs) can be used as prognostic markers and therapeutic targets in many cancers, including prostate cancer,^17^ lung adenocarcinoma,^18^ colorectal cancer,^19^ breast cancer,^20^ and liver cancer.^21^ The introduction of ICIs has significantly changed the treatment of HCC. Still, due to the complex pathogenesis of HCC and the immunosuppressive tumor microenvironment, the response rate to ICIs monotherapy is less than 20%, while the efficacy of combination therapy is about 30-36% and the development of immune resistance in some patients after successful treatment with ICIs is a major obstacle to immunotherapy for HCC.^22‐24^ Therefore, predicting HCC prognosis and therapeutic responsiveness based on combinations of ICIs using the LRGs model can help identify new therapeutic targets and strategies for HCC.

In our study, using transcriptomic data from the TCGA cohort, we stratified 368 HCC patients into two distinct subtypes based on LRGs. Comparative analysis revealed significant intergroup differences in biological pathways, tumor immune microenvironment (TIME) composition, somatic mutation profiles, and epigenetic modifications. Meanwhile, we identified three pivotal LRGs and developed a quantitative risk-scoring model. External validation in an independent cohort confirmed the model's robustness and predictive accuracy. These results demonstrate the strong prognostic potential of the model and its utility in guiding precision therapy for HCC patients.

## 2 Methods

### 2.1 Collection of sample information

HCC gene expression data and corresponding clinical information in this study were obtained from the publicly available TCGA and GEO databases. The FPKM values and clinical data for GDC TCGA Liver Cancer (TCGA-LIHC) were downloaded from the UCSC Xena database (https://xenabrowser.net/datapages/). The validation cohort GSE116174 was downloaded via the GEO database (https://www.ncbi.nlm.nih.gov/gds/). We excluded patients with missing survival information before the analyses and converted the expression data from FPKM values to TPM values uniformly. The ComBat method was used to correct for batch effects, and batch effects were validated using principal component analysis (PCA). Finally, we obtained TCGA-LIHC (*N* = 368) and GSE116174 (*N* = 64) datasets for analysis. The analytical workflow is presented in Figure S1, and the baseline clinicopathological features are detailed in Table S1.

### 2.2 Patient stratification via LRGs-based consensus cluster

We extracted 332 LRGs from previous studies^19^ and performed consensus clustering analysis using the ConsensusClusterPlus software package to identify unique patterns of transcriptional regulation associated with LRGs. This step was repeated 100 times to ensure reliability. The optimal number of clusters for K-means clustering was validated using the elbow method and silhouette coefficient, with the k value ranging from two to six and the parameter nstart set to 100.

### 2.3 TIME infiltration and immunophenoscore (IPS) analysis

We used the single sample gene set enrichment analysis (ssGSEA) algorithm to quantify the relative abundance of each cellular infiltration in HCC TIME. Twenty-eight immune cell subtypes were obtained from a previous study.^25^ Immune cell infiltration levels were quantified using ssGSEA enrichment scores. The IPS, a well-validated biomarker of the immunotherapeutic response, was employed to comprehensively evaluate both the intratumoral immune microenvironment and the tumor antigen presentation profile. A higher IPS represents better ICIs efficacy.^25^ Immune checkpoint-related gene sets were curated from published literature,^26^ encompassing three functional categories: major histocompatibility complex (MHC) molecules, immune co-stimulator(ICP) checkpoints, and immune co-inhibitory checkpoints (IAP).

### 2.4 Gene set variation analysis (GSVA)

We obtained “Hallmark gene sets” from the MSigDB database (https://www.gsea-msigdb.org/gsea/msigdb) and performed GSVA enrichment analysis using the “GSVA” R package. GSVA is a nonparametric, unsupervised approach that estimates pathway and biological process activity variations across transcriptomic samples. The resulting GSVA enrichment scores enable quantitative comparison of pathway-level differences between LRGs-defined molecular subtypes.

### 2.5 Mutation, methylation-driven genes, and copy number variation (CNV) analysis

The R package “maftools” was used to compare the somatic mutation data in the TCGA database and to analyze the significantly mutated genes. The differences between the mutation features obtained by cosine similarity extraction and the mutation database (the Cancer Somatic Mutation Catalogue database, COSMIC V2, https://cancer.sanger.ac.uk/signatures/signatures_v2/) were analyzed. Differential methylation-driven genes were identified by methylation data from the TCGA database (TCGA-LIHC Illumina HumanMethylation450) using the MethylMix software package. Significant differences in CNV fragments (alternation frequency >0.3) between the two LRGs isoforms were analyzed by waterfall and heatmap mapping using the “Complex Heatmap” R package.

### 2.6 Signature development and reliability evaluation

To establish prognostic associations, we first performed univariate Cox regression analysis to evaluate the relationships between LRGs mRNA expression and overall survival (OS) in HCC patients. Candidate mRNAs showing significant associations (*P* < 0.05) were subsequently analyzed using LASSO Cox regression followed by multivariate stepwise regression. The resulting regression coefficients were used to construct a lactylation-related genes signature (LRGS) model, which was calculated as: LRGS score = Exp1 * Coe1 + … + Expi * Coei, where Coei and Expi represent the regression coefficient and normalized expression value for each gene. Using the survminer R package, we stratified HCC patients into high-LRGS and low-LRGS groups based on optimal cutoffs derived from maximally selected rank statistics. Survival differences between groups were assessed by Kaplan‒Meier analysis with log-rank tests. The LRGS model's clinical utility was additionally validated in an independent GSE116174 cohort.

### 2.7 Predicting chemotherapy response and potential small-molecule drugs

The “oncoPredict​​”​​^27^ R package was utilized to predict the chemotherapeutic response in HCC patients. The half-maximal inhibitory concentration (IC50) of each patient was estimated through elastic net regression, a machine learning algorithm that combines both L1 (Lasso) and L2 (Ridge) regularization to optimize model generalizability. Model training and validation were performed using the Genomics of Drug Sensitivity in Cancer (GDSC, https://www.cancerrxgene.org/) database as the reference dataset. Potential small-molecule drugs for the treatment of HCC were identified through the Connectivity Map (Cmap, https://portals.broadinstitute.org/CMap/) database using the eXtreme Sum (XSum) algorithm.^28^

### 2.8 Validation of expression and immunohistochemical staining analysis

Differential expression analysis of the three LRGs in HCC was performed using TCGA data accessed through the UALCAN portal (https://ualcan.path.uab.edu).^29^ Protein-level validation was conducted by examining immunohistochemical staining results from the Human Protein Atlas database (HPA, https://www.proteinatlas.org/) for two LRGs.

### 2.9 Quantitative and statistical analyses

All the statistical analyses were conducted using R software (v4.5.0). For multigroup comparisons, we employed the Kruskal‒Wallis test with Dunn's post hoc correction. Prognostic stratification was achieved through consensus clustering of LRGs, with survival differences assessed by Kaplan–Meier analysis. Associations between molecular clusters and clinical characteristics were evaluated using Fisher's exact test. Univariate and multivariate Cox proportional hazards regression models were implemented to evaluate prognostic associations. *P* < 0.05 was considered a statistically significant difference. *P < 0.05, **P < 0.01, and ***P < 0.001.

## 3 Result

### 3.1 Two distinct HCC clusters mediated by LRGs

**Figure 1.**
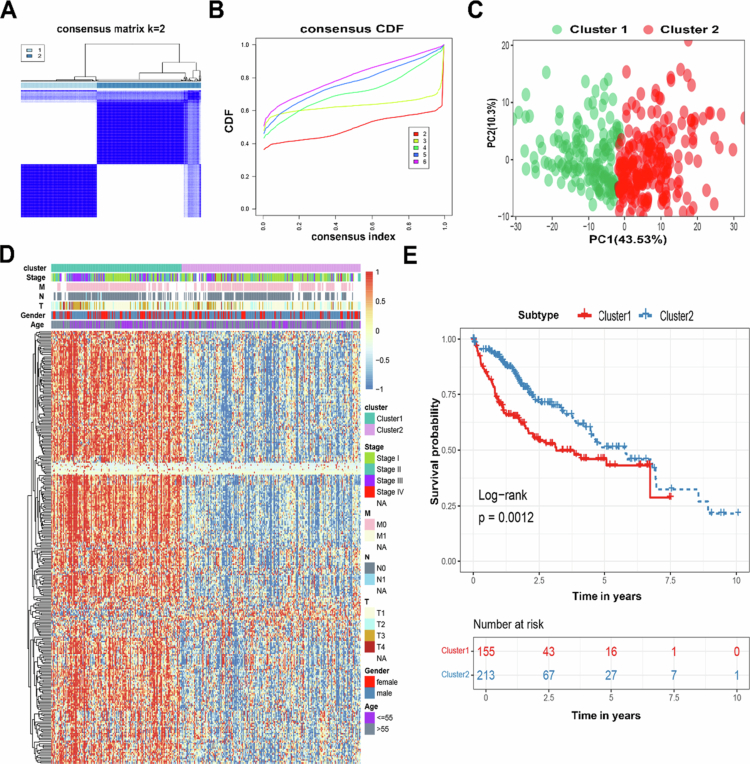
LRGs cluster analysis classified HCC patients into two distinct subtypes. (A and B) The optimal number of clusters (K = 2) was determined from the CDF curves, and the classification effect was the best. (C) PCA plot of HCC samples. (D) Heatmap analysis of LRGs expression and clinical features in the two subtypes. The expression of the LRGs was normalized to the Z-score. (E) Survival analysis of the two LRGs subtypes in the TCGA-LIHC cohort was created using Kaplan–Meier curves. *P < 0.05.

From published literature,^19^ we identified 332 LRGs and performed consensus clustering analysis on 368 TCGA-LIHC patients with complete survival data. This analysis stratified HCC patients into two robust subtypes: cluster 1 (*N* = 155) and cluster 2 (*N* = 213) (Figure 1A). To define the optimal number of molecular subtypes, we employed the elbow method and silhouette coefficient analysis. The within-cluster sum of squares (WCSS) exhibited a sharp decline before *k* = 2 and plateaued thereafter (Figure S2). Concurrently, the average silhouette coefficient reached its maximum at *k* = 2 (0.2130; Figure S3). Collectively, these results confirmed that *k* = 2 was the optimal clustering number for the current dataset. Cluster stability was validated through cumulative distribution function (CDF) analysis (Figure 1B). The clinical characteristics associated with the LRGs subtypes are presented in Table S1. PCA confirmed distinct transcriptional profiles between subtypes, validating the classification accuracy. We performed a clear subgroup division of the two clusters, which were different, as shown in Figure 1C. The heatmap results of the expression and clinical features of LRGs in HCC patients also showed significant differences in the expression of the two clusters (Figure 1D). From the perspective of survival analysis, it was observed that the OS rate of cluster 2 was higher than that of cluster 1, suggesting that LRGs-mediated isoforms have a prognostic value in HCC patients (*P* = 0.0012) (Figure 1E). These findings collectively establish LRGs as clinically significant prognostic biomarkers in HCC. In addition, we next compared the differences between the two subtypes in terms of their biological properties and prognostic characteristics (Figure S1).

### 3.2 Differential biological functions and TIME features of the two LRGs clusters

**Figure 2.**
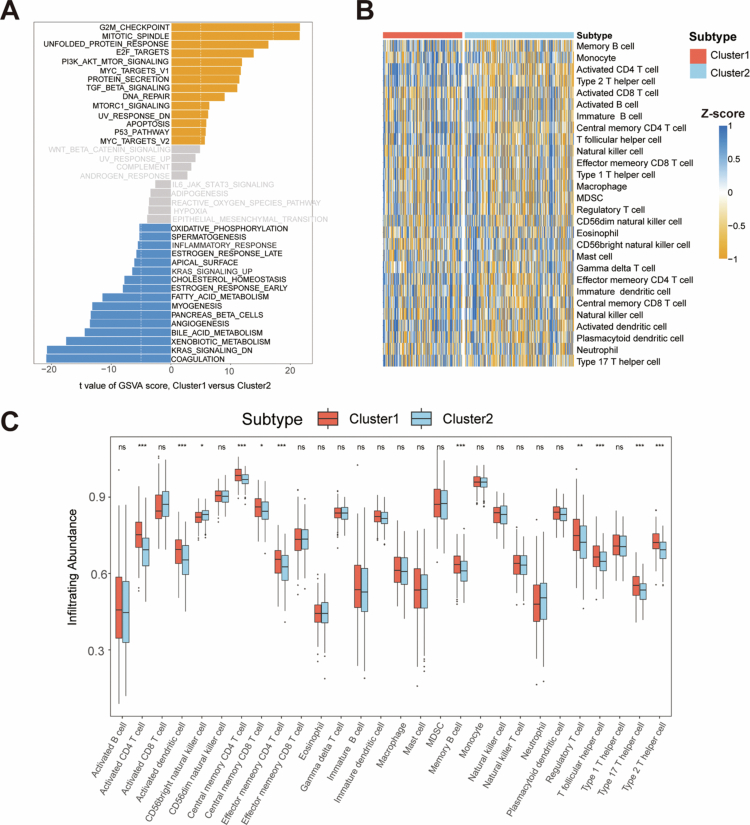
Biological function and TIME features of different LRGs clusters in the TCGA cohort. (A) The GSVA plot shows a differentially activated pathway between the two clusters. Yellow bars indicate activated pathways; blue bars indicate inactivated pathways (|*t*-value| > 5 and *P* < 0.05). (B) Heatmap of the abundance of 28 immune cell types in tumor-infiltrating immune cells within the HCC microenvironment between the two LRGs subtypes. (C) Box plot of the abundance of 28 immune cell types among the tumor-infiltrating immune cells in the HCC microenvironment between the two LRGs subtypes. The relative infiltration of each immune cell type was normalized to the Z-score. *P < 0.05, **P < 0.01, and ***P < 0.001.

Distinct biological functions and TIME features were observed between the two LRGs clusters. GSVA enrichment analysis revealed marked pathway activation differences: cluster 1 showed significant enrichment of proliferative pathways (mitotic spindle and G2/M checkpoint regulation), while cluster 2 demonstrated suppression of KRAS signaling alongside enhanced coagulation and xenobiotic metabolism pathways (Figure 2A). These findings were corroborated by both KEGG (Figure S4) and GO (Figure S5) analyses. Immune landscape characterization through 28-cell infiltration scoring revealed elevated overall immune activity in cluster 1 (Figure 2B). Specifically, cluster 1 exhibited significantly increased infiltration of 10 immune cell types, including activated CD4^+^ T cells, dendritic cells (DCs), and central memory CD4^+^ T cells (Figure 2C). These results establish LRGs as key regulators bridging tumor proliferation pathways, metabolic reprogramming, and immunomodulation in HCC.

### 3.3 Significant differences in the mutation patterns of the two LRGs clusters

**Figure 3.**
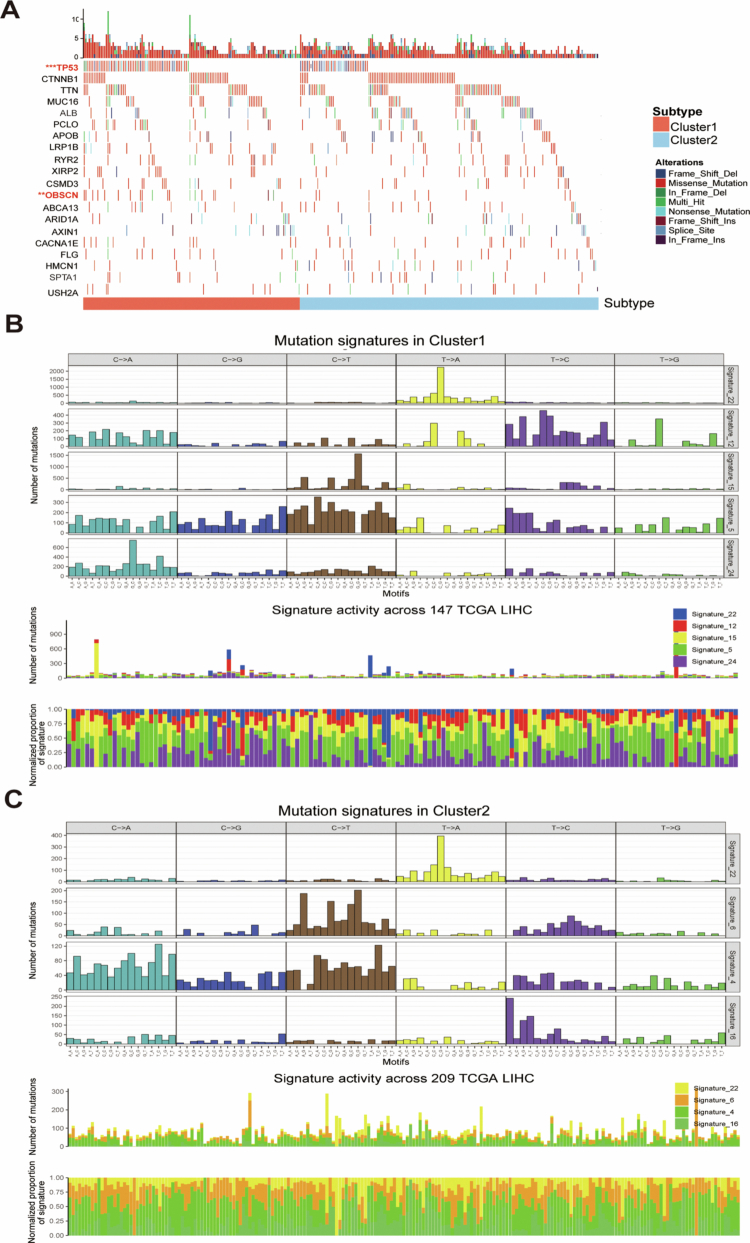
Mutational landscape in the two LRGs subtypes. (A) Mutational landscape of the genes between the two LRGs subtypes. (B) Mutation signatures in cluster 1. (C) Mutation signatures in cluster 2. *P < 0.05, **P < 0.01, and ***P < 0.001.

Comparative mutational analysis between the two subtypes and their relationship with LRGs, we first performed significant mutated genes (SMGs) analysis based on the TCGA-LIHC cohort. Waterfall plot visualization revealed significantly higher mutation frequencies of TP53 and OBSCN in cluster 1 compared to cluster 2 among the SMGs (Figure 3A). Based on the COSMIC V2 database revealed that the mutation manipulation process in cluster 1 was characterized by signature 5 and signature 24, while cluster 2 predominantly exhibited signature 4 and signature 16 (Figure 3B and C). Thus, the above results establish that LRGs-based classification effectively stratifies HCC patients into molecular subtypes with biologically distinct mutational landscapes.

### 3.4 Epigenetic differences between the two LRGs clusters

**Figure 4.**
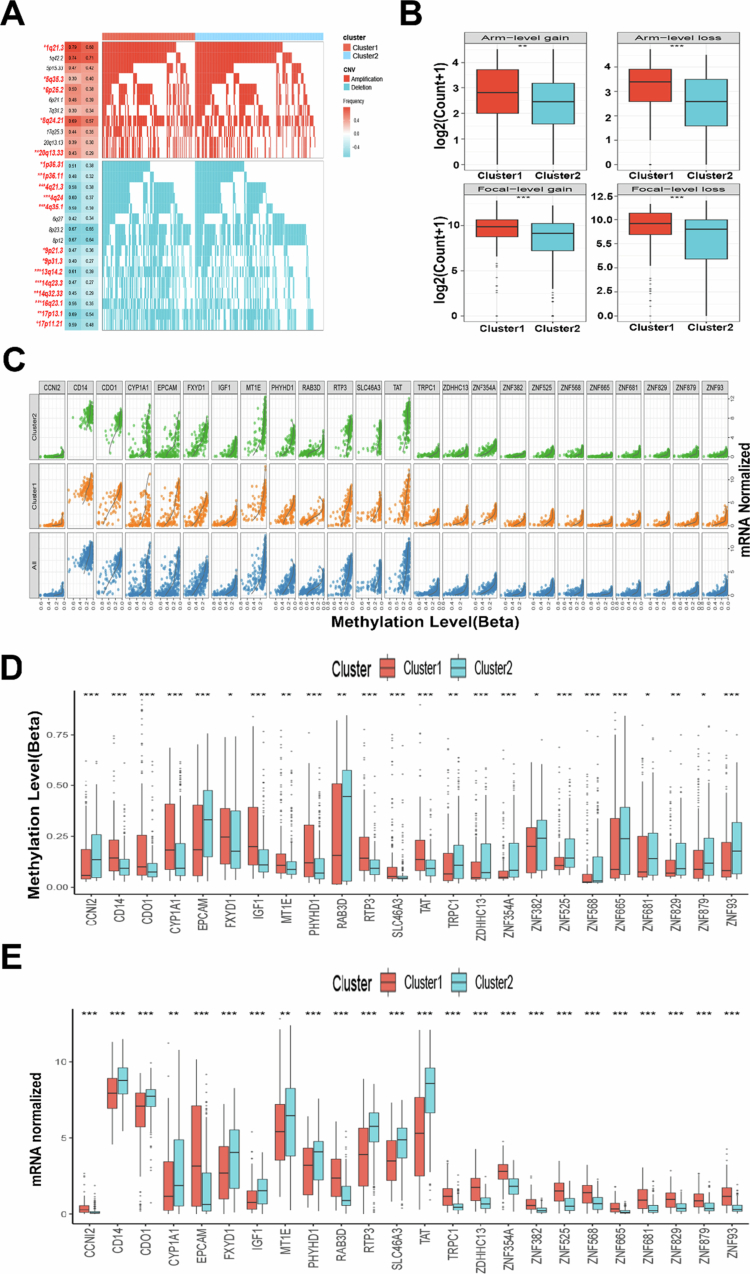
Epigenetic alterations in two clusters. (A) Detailed plots with copy number amplifications and deletions between the two LRGs subtypes. (B) Distribution of focal and broad copy number alterations (gain and loss) between the two LRGs subtypes. (C) The correlation of the beta value and gene expression based on 24 methylation-driven genes. (D and E) The Boxplot showing the methylation-driven genes in different LRGs subtypes. *P < 0.05, **P < 0.01, ***P < 0.001. The data are represented as mean (SD).

Characterizing the epigenetic landscape of malignant cells provides critical insights into oncogenic mechanisms and reveals novel therapeutic targets for intervention. First, we found that epigenetic landscapes differed substantially between LRGs subtypes, with significant CNV disparities. For example, cluster 1 had many copy number deletions (e.g., *1p36.31*, *1p36.11*, *4q21.3*, *4q35.1*, and *9p21.3*) and several copy number amplifications (e.g., *1q21.3*, *5q35.3*, *6p25.2*, *8q24.21*, and *20q13.33*) (Figure 4A). The cluster 1 exhibited a significantly greater overall CNV burden, with lesion-level and arm-level analyses demonstrating elevated frequencies of both copy number gains and losses (Figure 4B). Subsequently, MethylMix analysis identified 24 differentially methylated driver genes between the two clusters (Figure 4C), with corresponding expression changes (Figure 4E). These findings establish a robust association between LRGs expression and epigenetic regulation in HCC.

### 3.5 LRGS emerges as an independent prognostic biomarker for HCC

**Figure 5.**
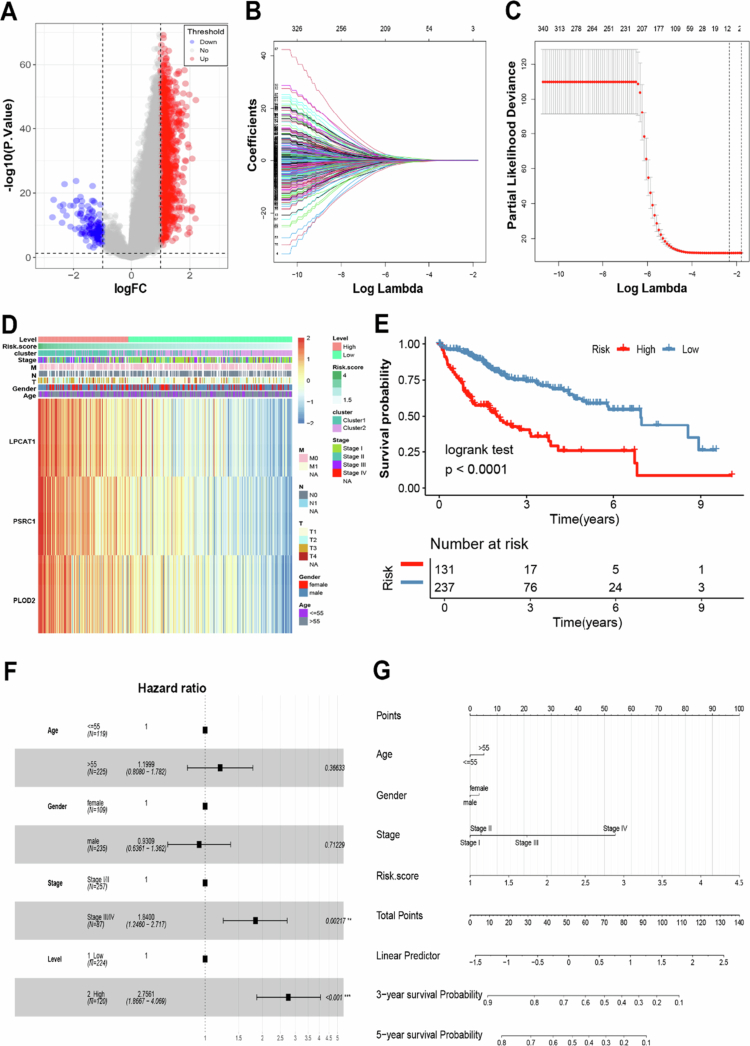
Identification of LRGS in HCC. (A) The volcano plot showing that 1181 differentially expressed mRNAs and 332 LRGs were between the cluster 1 and cluster 2 subtypes. The red dots indicate significant upregulation of mRNA expression, the blue dots indicate significant downregulation of mRNA expression, and the gray dots indicate no significant changes of mRNA expression. (B and C) LASSO variable screening process. (D) The expression of three mRNAs in HCC patients. (E) Survival analysis of LRGS in the TCGA-LIHC cohort was created using Kaplan–Meier curves. (F) Multivariate Cox regression analyses of the association between clinicopathological factors and OS of HCC patients in the TCGA-LIHC cohort. (G) The nomogram comprises clinical characteristics (age, sex, and stage) and the risk score. The variable scores were summed to obtain the total points, and the total point line is shown at the bottom of the nomogram. *P < 0.05, **P < 0.01, and ***P < 0.001.

Based on the above confirmation that HCC patients can be well classified into two categories based on LRGs, we constructed an LRGS capable of accurately quantifying the expression pattern scores of individual tumor patients. The procedure for constructing the LRGS was as follows: 1181 mRNAs from the TCGA-LIHC cohort were analyzed by univariate Cox regression, which were differentially expressed between the two LRGs subtypes (|log2FC| > 1 & *P* < 0.05), and the expression levels of significant genes in the two subtypes were represented by volcano plots (Figure 5A). Univariate and LASSO Cox regression analyses identified eight genes with significant prognostic value (Figure 5B and C). Stepwise multiple regression analysis revealed three core prognostic biomarkers (LPCAT1, PSRC1, and PLOD2) for LRGs-based HCC classification (Table S2). Additionally, the expression patterns of these genes correlated strongly with clinical features, with elevated expression in cluster 1 compared with cluster 2 (Figure 5D). We derived the LRGS prognostic signature using the formula: LRGS score = 0.2441 × exp (LPCAT1) + 0.2563 × exp (PSRC1) + 0.1892 × exp (PLOD2). This signature stratified patients into high-risk and low-risk groups (optimal cutoff = 2.599, determined by survminer), with significantly poorer survival in high-LRGS patients (log-rank *P* < 0.0001; Figure 5E). The model showed robust predictive accuracy (3-y AUC = 0.724; 5-y AUC = 0.699; Figure S6) and was validated in the GSE116174 cohort (Figure S7). Multivariate analysis confirmed the LRGS as an independent prognostic factor, with higher values associated with worse prognosis (Figure 5F).

In addition, the multivariate nomogram incorporating age, sex, tumor stage, and LRGS (Figure 5G) demonstrated robust prognostic performance. Calibration analysis revealed excellent agreement between the predicted and observed survival probabilities at both 3-y and 5-y timepoints (Figure S8). These results validate the LRGS as an effective stratification tool for distinguishing high-risk and low-risk HCC patients.

### 3.6 Analysis of the expression associated with the three LRGs

**Figure 6.**
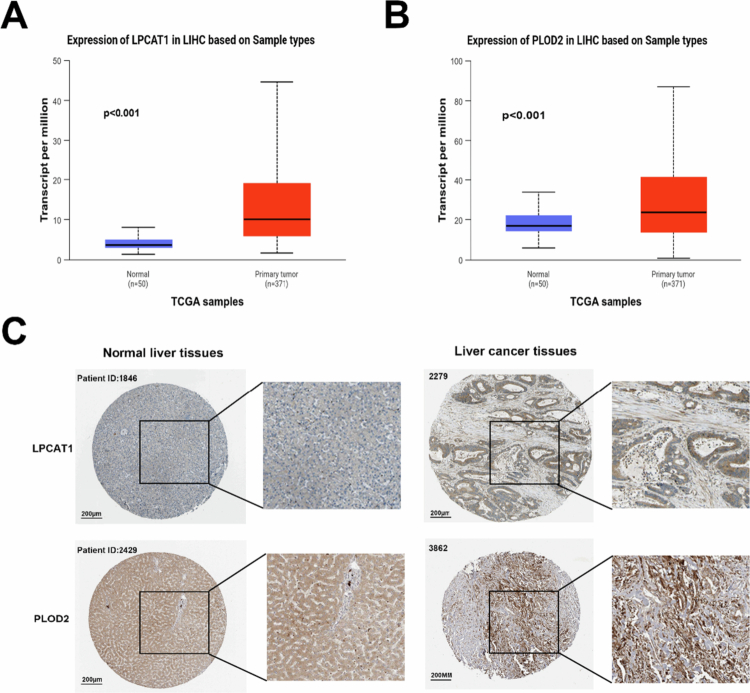
Correlation expression analysis of the LRGs. (A) Expression level analysis of LPCAT1 in HCC and normal tissues. (B) Analysis of the expression level of PLOD2 in HCC and normal tissues. (C) Immunohistochemical staining showing the protein expression of LPCAT1 and PLOD2 in normal liver tissues and liver cancer tissues from the HPA database.

To further investigate the differential expression of the three LRGs in the LRGS model in HCC patients, we analyzed the expression levels of LPCAT1, PSRC1, and PLOD2 in both HCC and normal tissues from the perspectives of gene expression and protein translation using UALCAN. The results showed that the relative expression levels of the three genes were significantly higher in HCC tissues than in neighboring normal tissues (Figures 6A, 6B, and S9). Immunohistochemical validation using the HPA database confirmed the corresponding protein-level overexpression of LPCAT1 and PLOD2 (Figure 6C), with no corresponding data for PSRC1.

### 3.7 LRGS stratification predicts chemotherapeutic response in HCC

**Figure 7.**
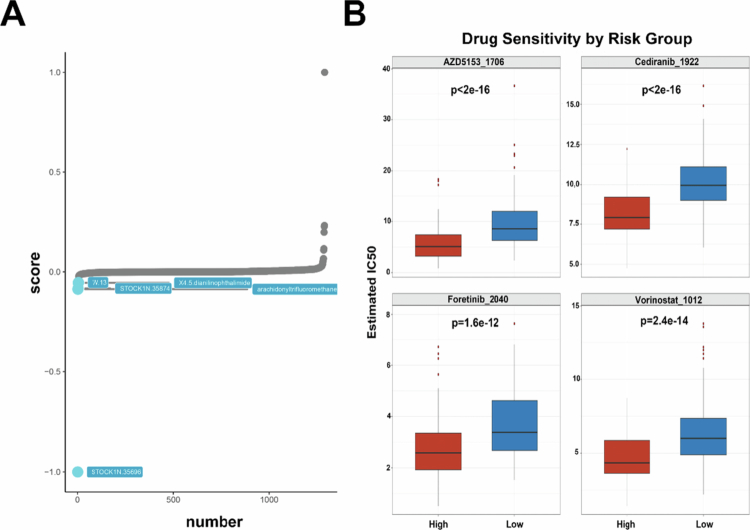
The predictive value of LRGS in chemotherapy drugs selection and responsiveness in the two risk groups. (A) The top 5 small-molecule compounds with LRGS. (B) IC50 estimates for AZD5153, cediranib, foretinib, and vorinostat in the high-LRGS and low-LRGS groups. The data are represented as mean (SD).

Given the key role of LRGs in HCC progression, we assessed the predictive value of LRGS in optimizing potential drug selection and drug treatment response in HCC patients. Using the XSum algorithm, we identified that the small-molecule drug STOCK1N.35696 was a potential therapeutic candidate for high-LRGS patients (Figure 7A). The prediction of drug response to chemotherapy for HCC by the “oncoPredict” found that the predicted IC50 values of the drugs AZD5153, cediranib, foretinib, and vorinostat were significantly lower in high-LRGS patients than in low-LRGS patients, suggesting that high-LRGS patients had a higher sensitivity to the above drugs (Figure 7B). These findings suggest that LRGS can be used to optimize appropriate chemotherapeutic agents for HCC patients.

### 3.8 Potential value of LRGS in immunotherapy

**Figure 8.**
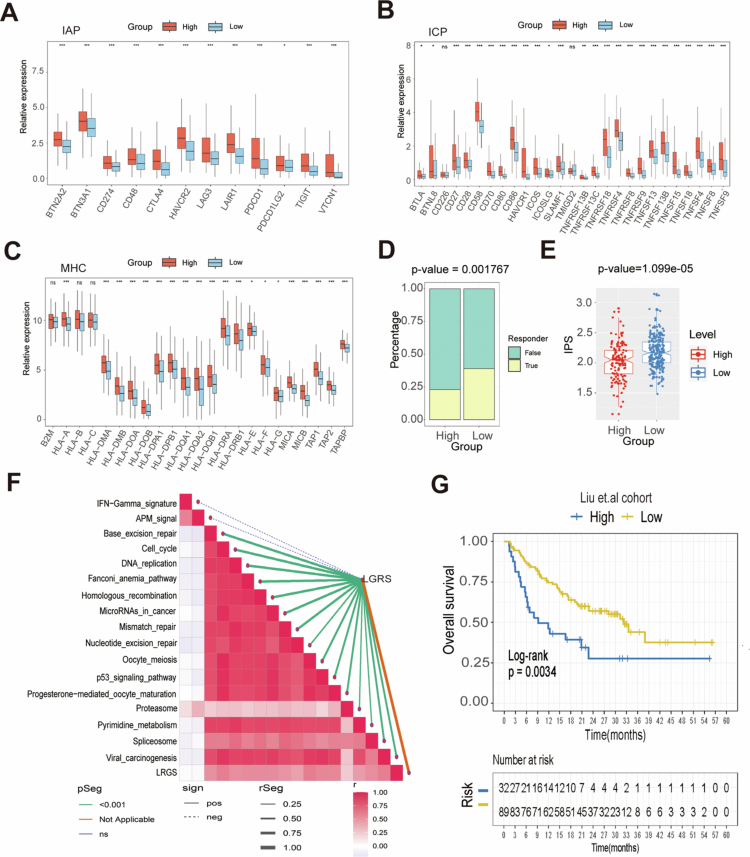
Prediction of response to immunotherapy by LRGS. (A–C) Expression levels of IAP, ICP, and MHC gene sets differed between the high-LRGS and low-LRGS groups. (D) The proportion of immunotherapy response rates was analyzed between the two groups by TIDE. (E) Boxplot of IPS in the high-LRGS and low-LRGS groups. (F) The correlation between the LRGS and immunotherapy-related pathways. (G) Kaplan‒Meier analysis of the high-LRGS and low-LRGS groups in the Liu et al. cohort. *P < 0.05, **P < 0.01, and ***P < 0.001. The data are represented as mean (SD).

Finally, we explored the predictive value of LRGS for immunotherapy in HCC patients. The relative expression of key immune checkpoint: IAP, ICP, and MHC (Figure 8A–C) molecules demonstrated higher levels of expression in the high-LRGS group, and lower levels of expression in the low-LRGS group. In addition, the results obtained using the Tumor Immune Dysfunction and Rejection (TIDE) tool provided strong support for the notion that patients with low-LRGS may have a good outcome with immunotherapy (Figure 8D). The IPS is created based on immune-related genes, and the higher the IPS score is, the better the outcome of the ICIs.^25^ The IPS analyses showed a high score for the low-LRGS group, as illustrated by the figure, while the high-LRGS group scored lower (Figure 8E). These findings suggest that patients in the low-LRGS group are more likely to have good outcomes in immunotherapy. In addition, correlation analysis between the LRGS and immunotherapy-related pathways supported the significant association between the LRGS and immunotherapy-related pathways (Figure 8F). Additionally, the LRGS demonstrated predictive value for immunotherapy response, as validated in an independent melanoma cohort.^30^ Kaplan‒Meier analysis revealed significantly improved OS in low-LRGS group patients (log-rank *P* = 0.0034; Figure 8F). These consistent findings establish LRGS as a promising biomarker for predicting both chemotherapeutic and immunotherapeutic responses across cancer types.

## 4 Discussion

HCC represents approximately 90% of primary liver malignancies and constitutes a significant global health burden. While ICIs have emerged as first-line therapy demonstrating survival benefits across multiple malignancies, their efficacy in advanced HCC remains limited.^31‐33^ The Warburg effect serves as one of the metabolic features of cancer cells, whereby tumor cells tend to accumulate more lactate than normal cells. This may lead to aberrant histone lactylation, which exacerbates genomic instability, promotes tumor growth, and confers resistance to tumor therapy.^34^ Furthermore, emerging evidence indicates that lactate-mediated protein lactylation, acting through metabolic sensors, interacts with proteomic alterations to drive oncogenic processes.^35‐37^ Previous studies, integrating multiomics analyses identified HCC subtypes of significance and revealed complex interactions in the tumor ecosystem during HCC development.^38^ For instance, Hao et al. constructed a prognostic risk model based on lipid metabolism-related genes that effectively correlates gene expression patterns with immune microenvironment characteristics.^39^ However, there is still a need for a systematic and comprehensive efficacy index to indicate the prognosis of HCC patients and the efficacy of ICIs. Our study was based on 332 LRGs, the 368 HCC patients from the TCGA database were classified into two optimal clusters, and cluster 2 had a better prognosis than cluster 1. Meanwhile, we further systematically analyzed the differences between the two clusters in terms of biological functions, TIME features, mutation patterns, and epigenetic changes and established a reliable LRGS model, which validated both the accuracy for prognosis prediction and the value for precision therapy.

In this study, we identified significant differences in TIME and biological properties of LRGs clusters using GSVA, indicating that G2M checkpoint signaling pathway and mitotic spindle were activated in cluster 1, whereas coagulation-related pathways and KRAS signaling downregulation were inhibited in cluster 2. This finding implies that G2M checkpoint signaling,^40^ mitotic spindle,^41^ KRAS signaling^42^ transmission and coagulation^43^-related pathways may be the key regulatory pathways of LRGs in HCC. Immunotherapy plays an important role in the treatment of HCC, which inhibits and eliminates tumor cells by stimulating specific immune responses, thereby reducing the rate of tumor recurrence and metastasis.^44‐46^ TIME is an integral component of immunotherapy and has received extensive attention, and TIME analysis contributes to immunotherapy responsiveness. Given the critical role of immune infiltration in tumor immunotherapy, we quantified the infiltration of 28 immune cell populations in the HCC microenvironment. Cluster 1 exhibited significantly elevated immune scores and higher infiltration levels of 10 specific immune cell types, including B cells, DCs, and distinct T cell subsets, compared to cluster 2. These findings demonstrate that LRGs substantially influence the TIME composition and immunomodulation in HCC, though further investigation is needed to elucidate the roles of noncellular components and their underlying mechanisms.

HCC is a highly heterogeneous cancer at the molecular and histological levels with unique somatic mutation patterns driving its development and progression, most commonly involving *TERT* promoter, *CTNB1*, and *TP53* mutations.^47^^,^^48^ We examined two SMGs, including *TP53* and *OBSCN*, in HCC patients of LRGs subtypes. *TP53* is the most commonly mutated gene in human cancers, and its mutations have a profound effect on tumor cell genome structure, expression, and clinical perspectives. *TP53* mutations in HCC also affect the expression of immune checkpoint molecules in cancer.^49^^,^^50^ *OBSCN* is also one of the common highly mutated genes in HCC.^51^ In addition, we extracted the mutation signatures based on the COSMIC database. The cluster 1 had independent signatures of signature 5 and signature 24, which were associated mainly with DNA damage, while the cluster 2 had independent signatures of signature 4 and signature 16, which might be related to smoking. Then, we investigated the different CNV alterations between the two LRGs subtypes. Previous studies on HCC showed that the copy number amplification of oncogenic driver genes CCND1, FGF19 (*11q13.2*) and VEGFA (*6p21*) was increased, while the tumor suppressor genes CDKN2A and CDKN2B (*9p21.3*) contained high-frequency deletions.^48^^,^^52^^,^^53^ We further found that the cluster 1 exhibited characteristic genomic alterations, including amplifications at *1q21.3*, *5q35.3*, *6p25.2*, *8q24.21*, and *20q13.33*, along with deletions at *1p36.31*, *1p36.11*, *4q21.3*, and *4q35.1*, demonstrating the highest overall CNV burden among subgroups. Furthermore, LRGs were associated with both direct methylation changes and subsequent expression modulation of methylation-regulated genes, as evidenced by 24 methylation-driven genes showing significant differential *β*-values and TPM expression (*p* < 0.05). These findings establish LRGs as key regulators of epigenetic reprogramming during HCC progression.

Additionally, comparative analysis revealed substantial molecular and clinical differences between LRGs-defined subtypes. The cluster 1 exhibited significantly poorer prognosis, elevated *TP53* mutation frequency, and increased chromosomal instability, as evidenced by higher arm-level and focal CNV burdens. These genomic alterations suggest enhanced DNA damage susceptibility in cluster 1. The TIME differed markedly between subtypes, with the cluster 1 showing increased infiltration of ten immune cell types. These multiomics differences collectively account for the observed prognostic disparity.

Through the above studies, we can find that LRGs have an important value in HCC classification. These findings underscore the need for an LRGS model as an independent prognostic biomarker to predict both HCC outcomes and ICIs therapeutic response. Here, we constructed an LRGS with three mRNAs (LPCAT1, PSRC1, and PLOD2) obtained by multiple regression analysis. Among them, LPCAT1 functioned as a novel prognostic molecular marker in HCC, and its elevation was associated with poor prognosis and accelerated progression in patients by promoting cell growth, migration, and metastasis.^54^^,^^55^ PSRC1, a downstream effector of TP53, functions as a putative oncogene in HCC pathogenesis.^56^ PLOD2 has emerged as an independent prognostic factor for both OS and RFS in HCC, with mechanistic studies implicating its crucial role in tumor invasion and metastatic progression.^57^^,^^58^ In addition, transcriptomic and proteomic validation was performed using the UALCAN and HPA databases, confirming the differential expression patterns and subcellular localization of these three genes between HCC and normal tissues. Unfortunately, the HPA database lacked immunohistochemical analysis of PSRC1. However, the above results can better prove that LRGs play a key role in HCC carcinogenesis, and our LRGS model—constructed based on the three key LRGs—has certain reference value. Meanwhile, we validated TCGA-LIHC patients accordingly, and the results showed that the survival rate of the high-LRGS group was significantly lower than that of the low-LRGS group, and the calibration plots of 3-y and 5-y OS were well predicted.

To evaluate the clinical translation potential of the LRGS, we used the XSum algorithm and the TIDE tool to explore the relationship between the LRGS and clinical treatment. The results showed that high-LRGS patients had benefits in the small-molecule drug STOCK1N.35696. For the antineoplastic drugs AZD5153, cediranib, foretinib, and vorinostat, the high-LRGS group showed lower estimated IC50 values. These findings suggest potential therapeutic applications for HCC, though clinical validation remains necessary. IPS analysis revealed enhanced ICIs' efficacy in low-LRGS patients. Validation in an independent immunotherapy cohort^30^ confirmed superior OS in low-LRGS patients. The LRGS model thus demonstrates robust predictive value for both HCC prognosis and ICIs response, with potential applicability across cancer types.

**Figure 9.**
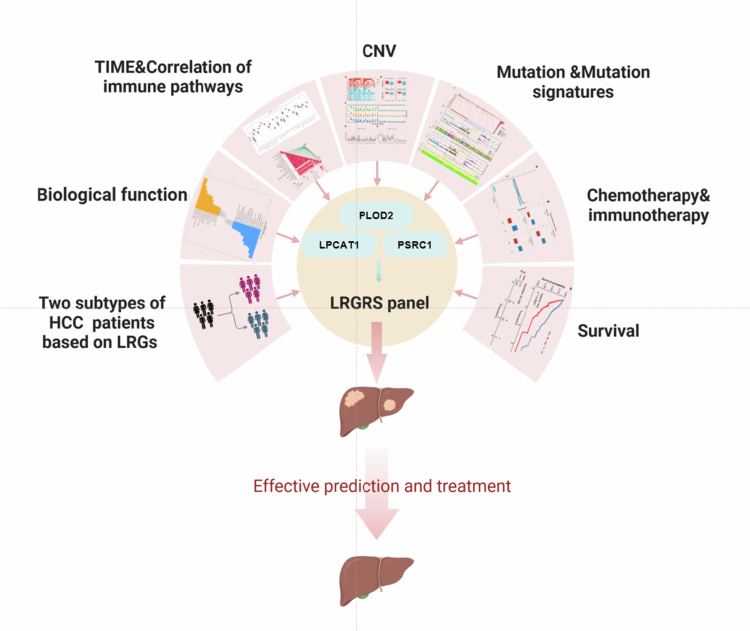
Experimental technical roadmap. (A) Adapted from “Mechanisms of Cancer-associated Fibroblast Activation” by BioRender.com (2025). Retrieved from https://app.biorender.com/biorender-templates.

Overall, we systematically identified the differences in biological pathways, TIME features, mutation patterns, genetic, and methylation changes between the two LRGs clusters. The LRGS model effectively stratified HCC patients into distinct prognostic groups, with high-LRGS patients demonstrating significantly worse OS. In addition, we found that the LRGS model can screen patients with HCC for better chemotherapeutic and immunotherapeutic agents and validated the scoring model in the Liu et al. immunotherapy cohort. However, we currently lack specific clinical data and experimental validation, limiting further interpretation of the findings. Future studies should address these limitations and require further in vivo and in vitro studies to optimize clinical therapeutic strategies by combining appropriate experimental models and clinical data for targeted studies of lactic acidification and immune pathways in HCC. Taken together, we developed an LRGS model that robustly predicts clinical outcomes and therapeutic responses in HCC patients. This model demonstrates significant potential for clinical translation, enabling simultaneous assessment of prognosis, chemotherapy sensitivity, and ICIs (Figure 9).

## 5 Conclusion

We classified HCC patients into two clusters based on LRGs and systematically analyzed the distinctions between the two clusters in terms of biological function, TIME features, mutation patterns, genetic, and methylation changes, and established an LRGS model for HCC risk assessment, which provides practical drug responsiveness evaluation for clinical treatment. However, we currently lack specific clinical data and experimental validation, limiting further interpretation of the findings, which still need to be validated, as well as larger cohorts to be effective. In summary, our constructed LRGS is expected to be an effective tool for predicting clinical outcomes and chemotherapeutic efficacy in HCC patients and to meet the requirements of clinical HCC treatment to some extent.

## Supplementary Material

Supplementary Material.docx Supplementary Material.docx

## Data Availability

All data generated or analyzed during this study are included in this published article.
