# Supplementary material for: Lactylation-related genes signature panel in hepatocellular carcinoma reveals the prognostic and therapeutic optimization
Source: Mol Cell Oncol. 2026 Feb 22;13(1):2621475. doi: 10.1080/23723556.2026.2621475 (PMC12928659; doi:10.1080/23723556.2026.2621475)
Supplement: Supplementary Material.docx [file KMCO_A_2621475_SM6552.docx]

**Supplemental Information**

**Supplemental information titles and legends**

**Figure S1.** The workflow of our study.

**Figure S2.** The result of GSEA (KEGG) analysis between two LRGs subtypes.

**Figure S3.** The result of GSEA (GO) analysis between two LRGs subtypes.

**Figure S4.** The ROC curves to predict predict the 1-years, 3-years and 5-years survival rate of LRGS in TCGA cohort.

**Figure S5.** Kaplan-Meier analysis of high-LRGS and low-LRGS groups survival probability in the GEO cohort.

**Figure S6.** The calibration plots for the 3-years and 5-years OS predictions in the TCGA cohort.

**Figure S7.** Analysis of PSRC1 Expression Levels in HCC and Normal Tissues.

**Table S1.** The relationship between LRGs subtypes and clinical indexes in TCGA-LIHC cohort (N = 368).

**Table S2.** Stepwise multiple regression analysis results.

**Figure S1**

**
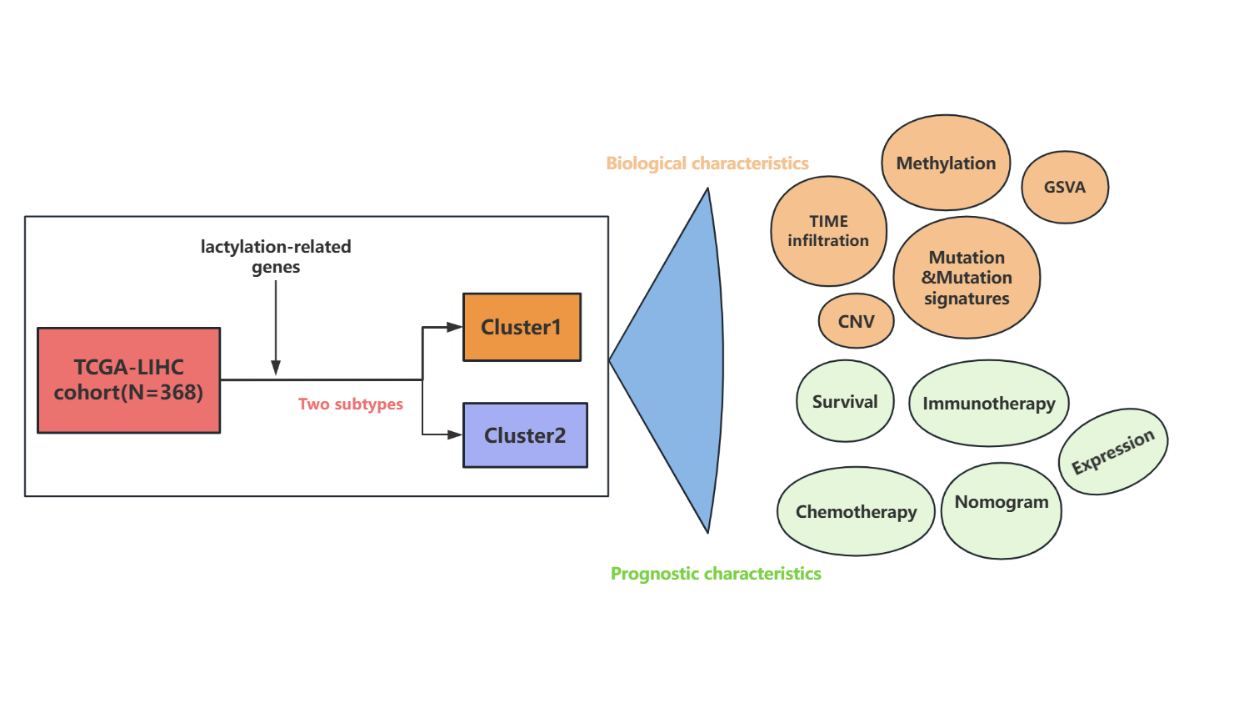
**

**Figure S1.** The workflow of our study.

**Figure S2**

**
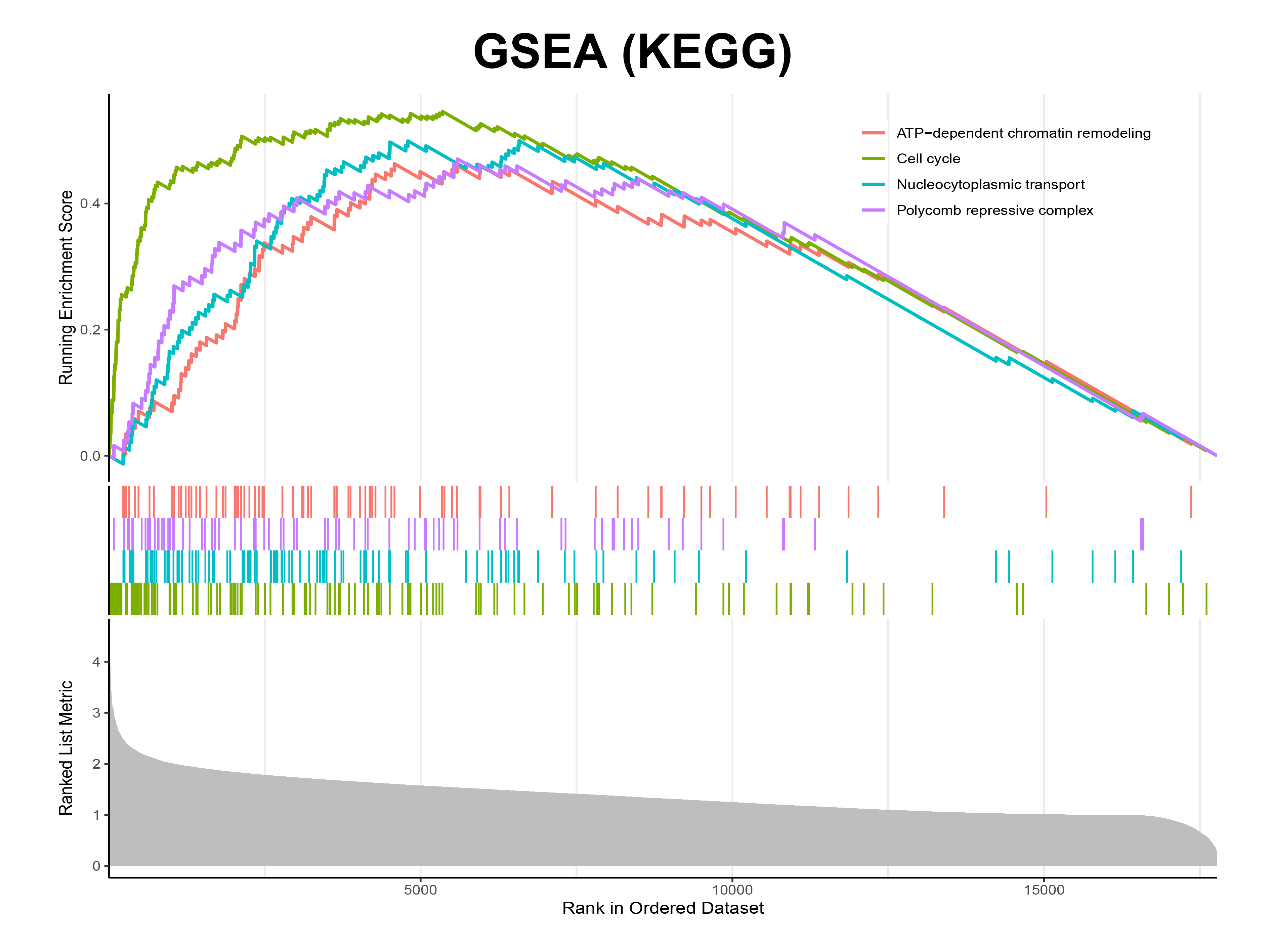
**

**Figure S2.** The result of GSEA (KEGG) analysis between two LRGs subtypes.

**Figure S3**

**
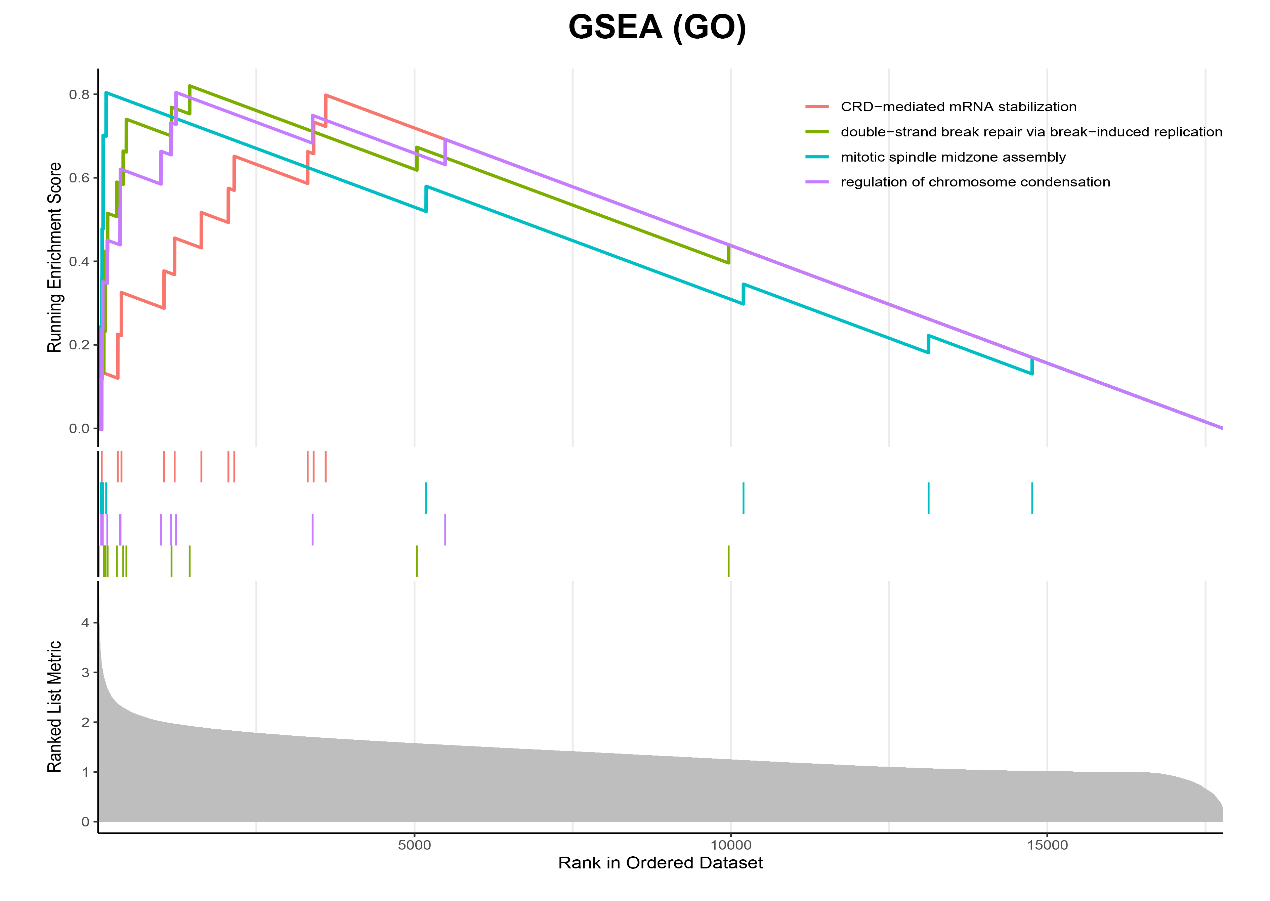
**

**Figure S3.** The result of GSEA (GO) analysis between two LRGs subtypes.

**Figure S4**

**
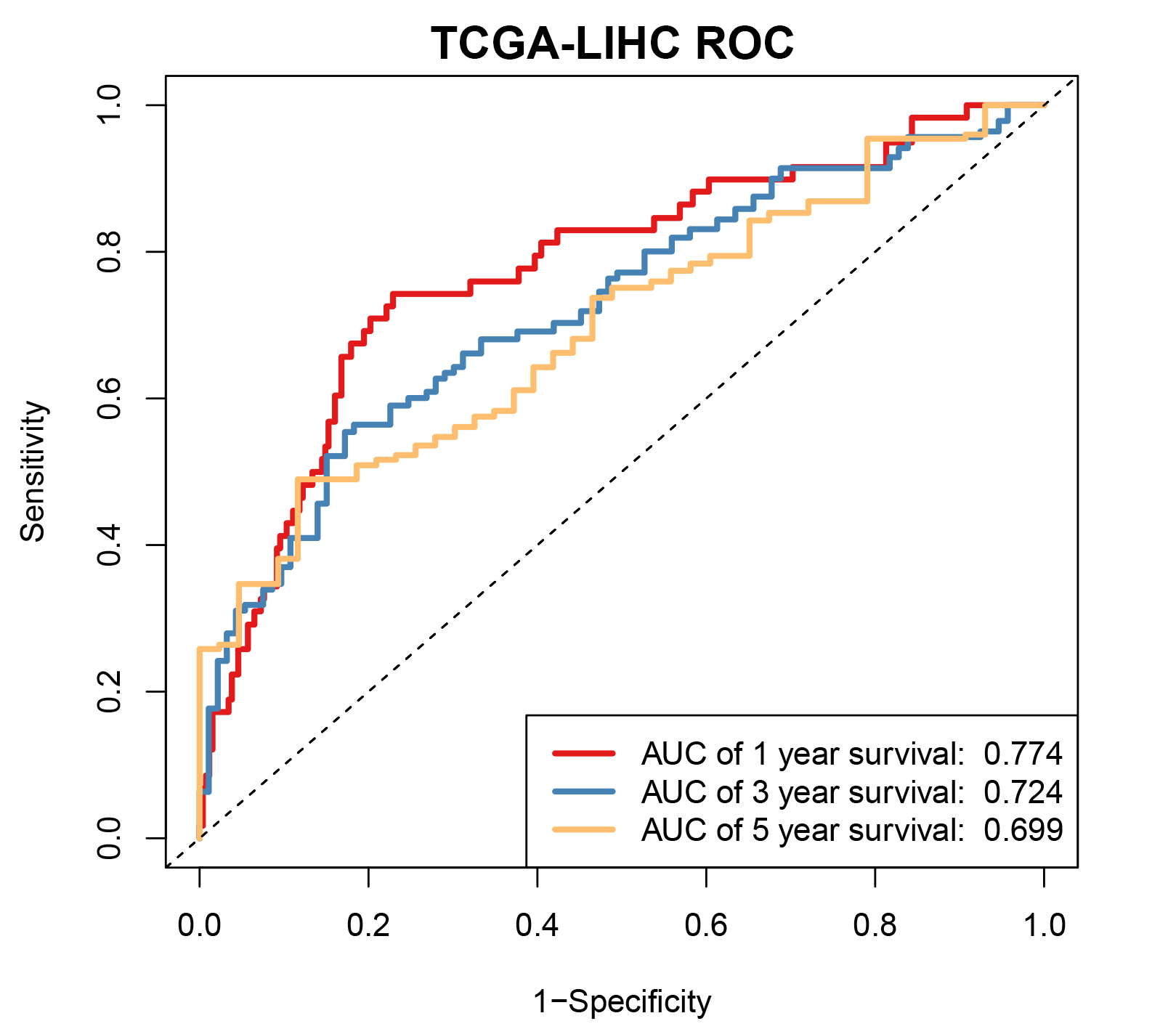
**

**Figure S4.** The ROC curves to predict predict the 1-years, 3-years and 5-years survival rate of LRGS in TCGA cohort.

**Figure S5**

**
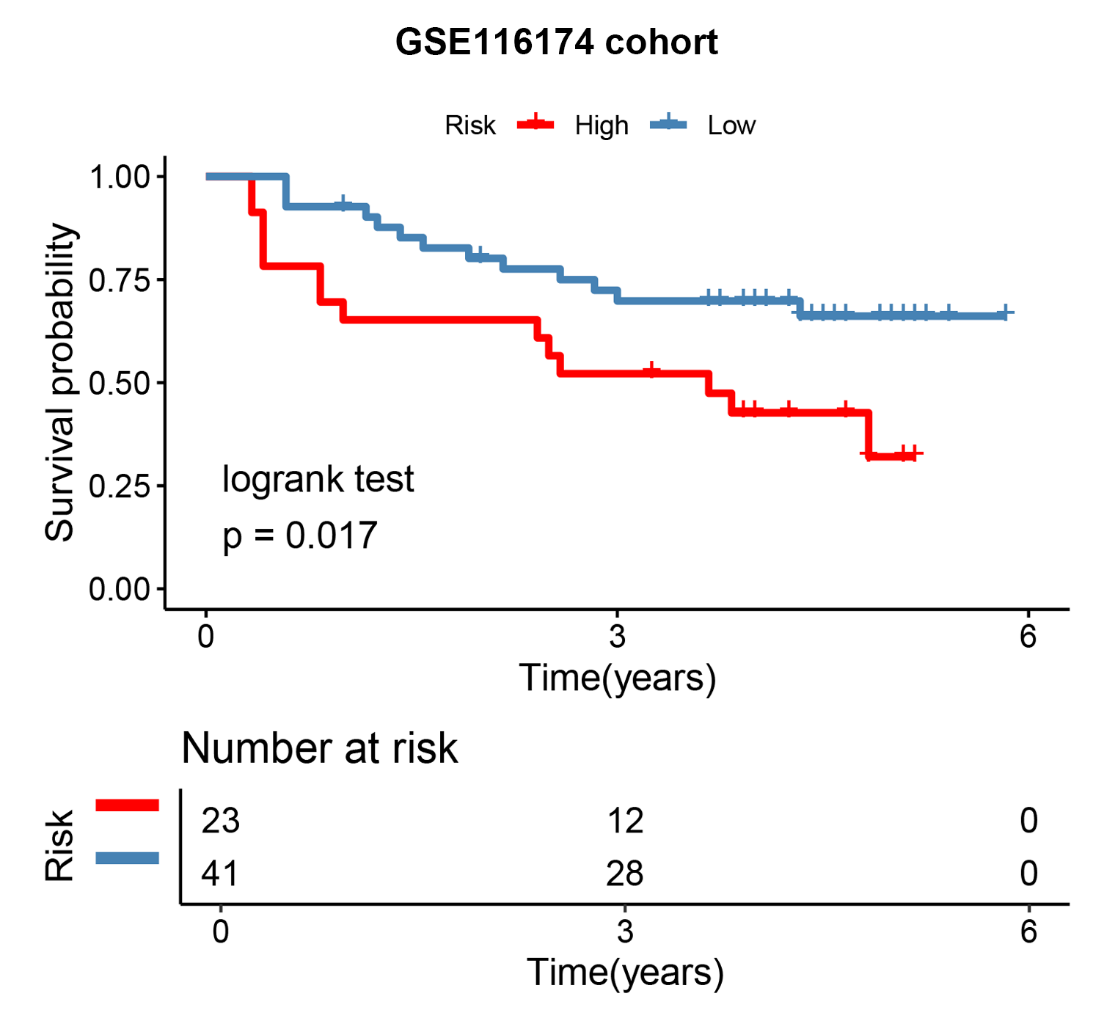
**

**Figure S5.** Kaplan-Meier analysis of high-LRGS and low-LRGS groups survival probability in the GEO cohort.

**Figure S6**

**
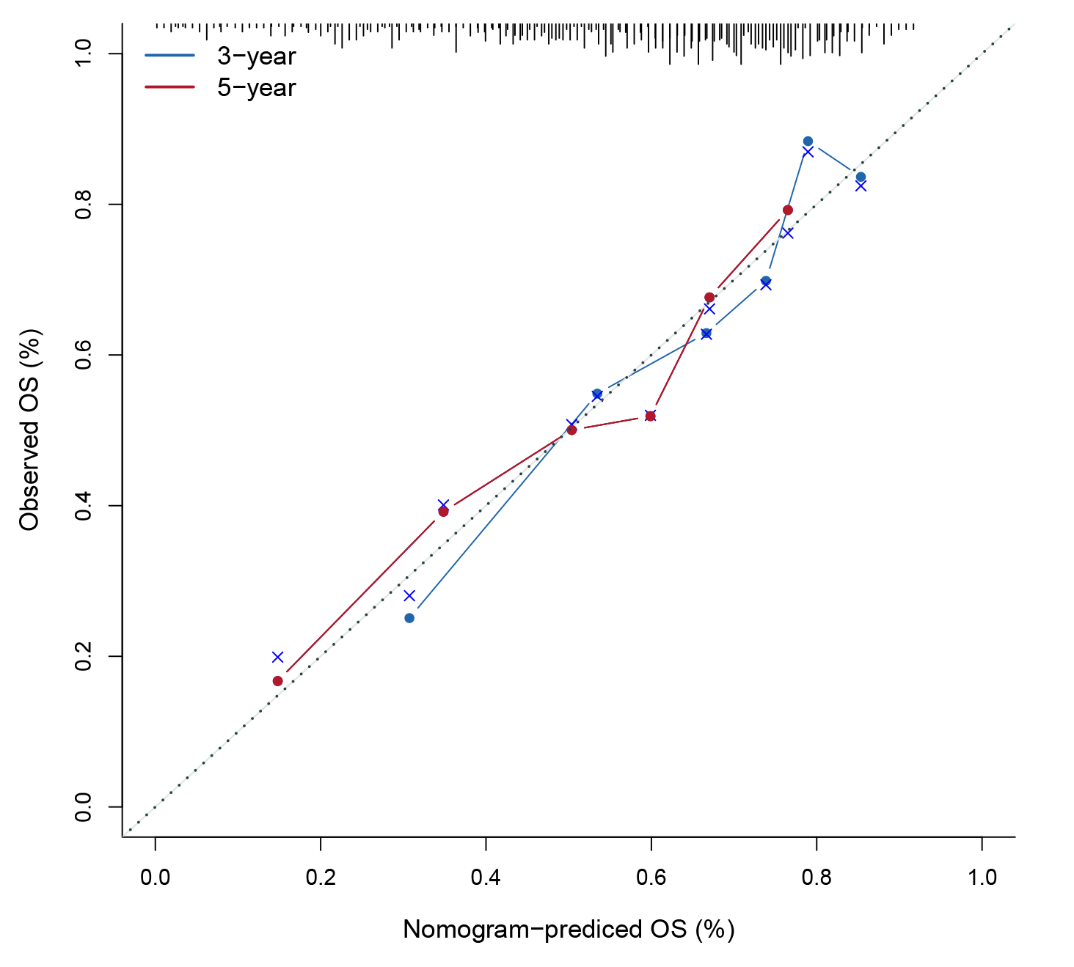
**

**Figure S6.** The calibration plots for the 3-years and 5-years OS predictions in the

TCGA cohort.

**Figure S7**

**
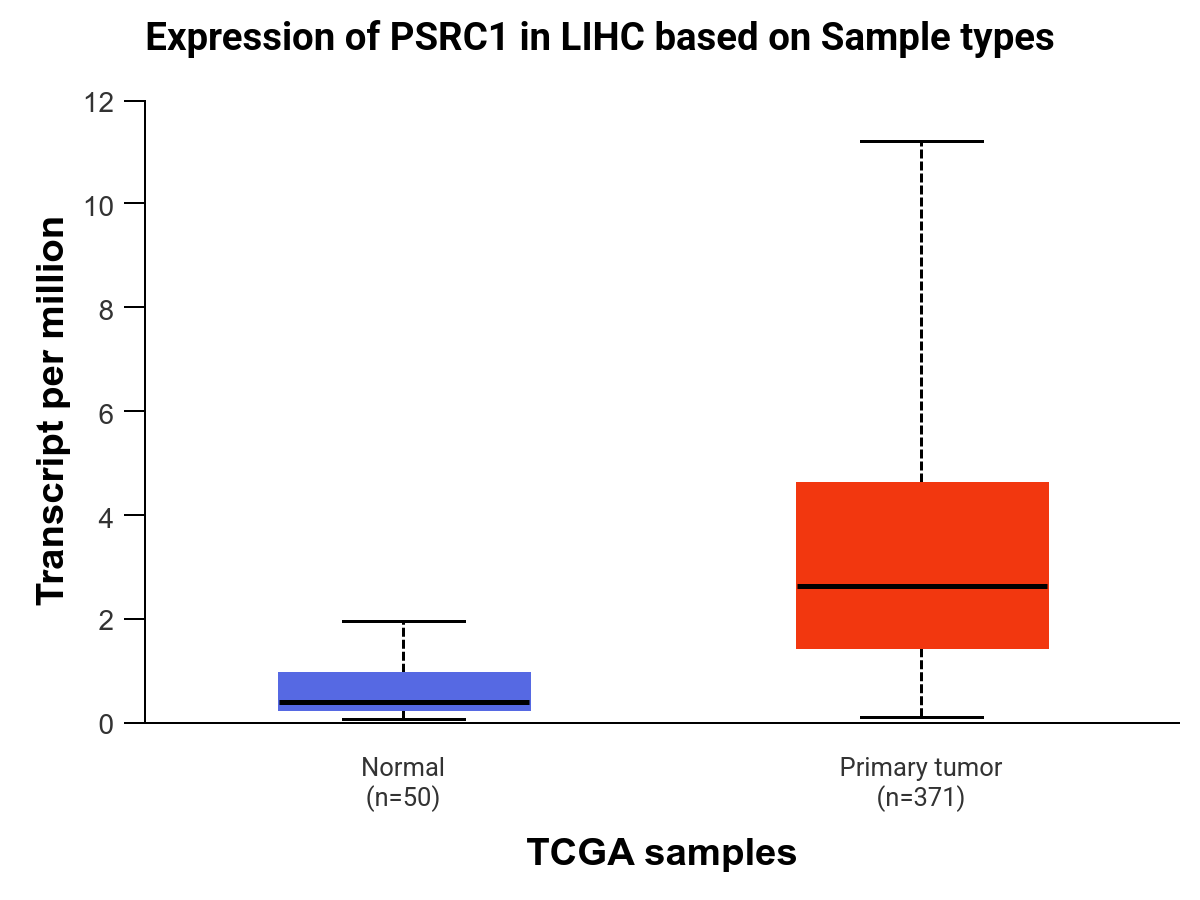
**

**Figure S7.** Analysis of PSRC1 Expression Levels in HCC and Normal Tissues.

**Table S1.** The relationship between LRGs subtypes and clinical indexes in TCGA-LIHC cohort (*N* = 368).

| **TCGA** | **Total(n=368)** | **Cluster1(n=155)** | **Cluster2(n=213)** | ***P*** |
| --- | --- | --- | --- | --- |
| **Age** |  |  |  | 0.0336^a^ |
| ≤55 | 122 | 61 | 61 |  |
| ＞55 | 246 | 94 | 152 |  |
| **Gender** |  |  |  | 0.3099 |
| Female | 119 | 55 | 64 |  |
| Male | 249 | 100 | 149 |  |
| **TNM stage** |  |  |  | 0.000426^a^ |
| Stage Ⅰ | 172 | 59 | 113 |  |
| Stage Ⅱ | 85 | 39 | 46 |  |
| Stage Ⅲ | 83 | 49 | 34 |  |
| Stage Ⅳ | 4 | 0 | 4 |  |
| NA | 24 | 8 | 16 |  |

^a^ Indicate statistically significant (Fisher's Exact Test, *P* < 0.05)

**Table S2.** Stepwise multiple regression analysis results.

| **Gene** | **Coef** | **Exp(coef)** | **Ee(coef)** | **z** | **Pr(>\|z\|)** | **Lower .95** | **Upper .95** |
| --- | --- | --- | --- | --- | --- | --- | --- |
| ***PSRC1*** | 0.2563 | 1.2921 | 0.1069 | 2.3976 | 0.0165 | 1.0479 | 1.5933 |
| ***PLOD2*** | 0.1892 | 1.2083 | 0.0884 | 2.1417 | 0.0322 | 1.0162 | 1.4368 |
| ***LPCAT1*** | 0.2441 | 1.2765 | 0.0754 | 3.2387 | 0.0012 | 1.1012 | 1.4797 |
